# Reviewed article: Melo MS, Souza TTCM, Soares LM, Santos AD, Raiol T, Ribeiro A. Human papillomavirus vaccination access, coverage and dropout in the Federal District, Brazil: a time series study, 2013-2023. Epidemiol Serv Saude 2025;34:e20240006. 10.1590/S2237-96222025v34e20240006.en

**DOI:** 10.1590/S2237-96222025v34e20240006.a

**Published:** 2025-04-07

**Authors:** Andréa Tenório Correia da Silva

**Affiliations:** 1 Faculdade de Ciências Médicas da Santa Casa de São Paulo, São Paulo, SP, Brazil

## Peer review A

## Questionnaire

Does the manuscript contain new and significant information to justify publication? Yes

Does the Abstract (Summary) clearly and accurately describe the content of the article? Yes

Is the problem significant and concisely stated? Yes

Are the methods described comprehensively? Yes

Are the interpretations and conclusions justified by the results? Yes

Is adequate reference made to other work in the field? Yes

## Manuscript Structure

Length of article is: Too long

Number of tables is: Adequate

Number of figures is: Adequate

**Please state any conflict(s) of interest that you have in relation to the review of this paper (state “none” if this is not applicable).**

None

**Interest:** Excellent

**Quality**: Average

**Originality:** Good

**Overall:** Good

**Recommendation:** Major Revision

## Comments

Comentário geral sobre o manuscrito
1.1 Os autores realizaram pesquisa sobre tema atual e relevante para o Sistema Único de Saúde (SUS). O estudo ecológico de série temporal realizado teve como objetivo analisar a tendência temporal de acesso, cobertura e abandono da vacinação contra o HPV em adolescentes, no período de 2013 a 2023, no Distrito Federal. Os autores observaram diferenças significativas entre os sexos: enquanto no sexo feminino houve uma tendência crescente para o abandono e decrescente para acesso e cobertura, o sexo masculino apresentou redução do abandono e aumento do acesso e da cobertura.
1.2 Contribuição para o SUS.
A pesquisa fornece dados epidemiológicos sobre acesso, cobertura e abandono da vacinação contra HPV em adolescentes no período de 2013 a 2023, o que inclui o período de pandemia da COVID-19. Esses dados podem contribuir para elaboração de estratégias relacionada à efetividade da implementação da vacinação contra o HPV em adolescentes.
Pontos do manuscrito que precisam ser aprimorados ou descrito com maior clareza:
2.1 Introdução. O arcabouço teórico descrito na introdução não inclui tópicos essenciais em pesquisas sobre cobertura vacinal:
(a) A importância das equipes de Atenção Primária à Saúde (APS), sendo o Brasil com um dos maiores programas de APS do mundo, sendo referência para outros países de baixa e média rendas;
(b) Descrição de barreiras e facilitadores para acesso e cobertura;
(c) Fatores associados ao abandono;
(d) Impactos dos marcadores sociais da diferença sobre o acesso à vacinação, incluindo sexo, raça/cor, local de moradia;
(e) Importância da desinformação em saúde e disseminação de notícias falsas para a hesitação vacinal, com aumento exponencial durante a pandemia da COVID-19;
(f ) Incluir a explicação da importância do estudo ser realizado no DF, centro urbano com grande desigualdade social, a descrição da variação do IDH nas regiões e dos dados referentes ao câncer de colo em comparação a outras cidades, grandes centros urbanos no Brasil.
(g) Descrever claramente o que o estudo agrega à literatura existente sobre o tema e qual a lacuna do conhecimento que a pesquisa se propõe a preencher.
2.2 Método
(a) Os autores analisaram o período de 2013 a 2023, o que inclui o período da pandemia da COVID-19, de grave crise sanitária, com alterações nas dinâmicas de vida, incluindo distanciamento social com medida para enfrentamento da pandemia, que associado ao aumento exponencial da demanda nos serviços de na APS, que priorizaram o atendimento aos usuários com suspeita ou diagnóstico de COVID-19, podem ter reduzido o acesso às salas de vacina das unidades básicas de saúde e, consequentemente, ter impactando os desfechos investigados. Minha sugestão é analisar os dados comparando dois períodos: pré-pandemia e durante a pandemia.
(b) Os períodos analisados para participantes do sexo feminino são diferentes daqueles analisados para o sexo masculino, o que dificulta a comparação. É necessário padronizar os mesmos períodos de comparação entre os grupos.
(c) O acesso às equipes de APS pode afetar o acesso, a cobertura e o abandono. Como se caracteriza a cobertura da APS regiões do DF analisadas? Há diferença entre os desfechos de acordo com cobertura de APS entre as regiões do DF?
(d) A variável raça/cor dos adolescentes não foi analisada. Sugiro verificar se há diferença entre os desfechos em relação a variável raça/cor? Analisar essa diferença pode contribuição para identificar inequidades e guiar a elaboração de estratégias; essa análise pode indicar vulnerabilidades sobrepostas e orientar as práticas de saúde na perspectiva interseccional.
Resultados
(a) Na tabela 1, os números referentes ao sexo masculino dos anos 2015 e 2016 estão discrepantes em relação aos outros anos. Verificar a consistência dos dados e justificar essa diferença.
Discussão
(a) A estrutura da seção discussão está extensa. A integração entre os parágrafos e a coesão do texto pode ser aprimorada.
(b) Uma sugestão é iniciar a seção discussão destacando, no primeiro parágrafo, os principais achados, respondendo à sua principal pergunta de pesquisa e enaltecendo a novidade do estudo.
(c) Os autores apresentam como um dos principais achados as diferenças encontradas nos desfechos entre adolescentes do sexo feminino e do sexo feminino. Entretanto, não discutem o tema em maior profundidade, não apresentam hipóteses que possam contribuir para explicar a diferença. Aprofundar essa discussão é representa uma contribuição importante do manuscrito.
(d) Nos primeiros quatro parágrafos, algumas informações estão dispostas de forma redundante. Sugiro revisão dos parágrafos e disposição das informações descritivas de forma concisa.
(e) Na página 13 na linha 39, os autores descrevem brevemente alguns fatores que pode constituir barreira para a cobertura como “exposição a rumores e mitos”. Sugiro ampliar a discussão sobre desinformação em saúde, disseminação de notícias falsas sobre vacinas, em particular durante a pandemia da COVID-19, pode afetar a hesitação e recusa vacinal.
(f) É necessário descrever os impactos dos achados para as práticas de saúde em particular para as equipes de APS, uma vez que a vacinação compõe o escopo do trabalho dos profissionais da APS no SUS.
(g) Sobre as limitações do estudo, não há descrição sobre dados ignorados ou campos em branco, que medidas os autores aplicaram para minimizar vieses. Explicar a não inclusão da variável raça/cor na análise.
(h) Sugiro que os autores descrevam quais são as forças do estudo.

